# Motor demands influence conflict processing in a mouse-tracking Simon task

**DOI:** 10.1007/s00426-022-01755-y

**Published:** 2022-11-20

**Authors:** Victor Mittelstädt, Hartmut Leuthold, Ian Grant Mackenzie

**Affiliations:** Department of Psychology, University of Tübingen, Schleichstraße 4, 72076 Tübingen, Germany

**Keywords:** Simon effect, Conflict task, Cognitive control, Motor control, Delta plots, Mouse-tracking

## Abstract

Previous studies have shown incorrect motor activation when making perceptual decisions under conflict, but the potential involvement of motor processes in conflict resolution is still unclear. The present study tested whether the effects of distracting information may be reduced when anticipated motor processing demands increase. Specifically, across two mouse-tracking Simon experiments, we manipulated blockwise motor demands (high vs. low) by requiring participants to move a mouse cursor to either large versus small (Experiment 1) or near versus far (Experiment 2) response boxes presented on the screen. We reasoned that participants would increase action control in blocks with high versus low motor demands and that this would reduce the distracting effect of location-based activation. The results support this hypothesis: Simon effects were reduced under high versus low motor demands and this modulation held even when controlling for time-varying fluctuations in distractor-based activation via distributional analyses (i.e., delta plots). Thus, the present findings indicate that anticipation of different motor costs can influence conflict processing. We propose that the competition between distractor-based and target-based activation is biased at premotor and/or motor stages in anticipation of motor demands, but also discuss alternative implementations of action control.

Goal-directed behavior requires action control, the ability that enables us to translate action-relevant information into appropriate motor responses (e.g., Verbruggen et al., 2014; Shiffrin & Schneider, 1977). Central to our understanding of action control is the key question of how decision-making and motor processes interact to optimize sensorimotor behavior (e.g., Wispinski et al., 2018; Kim et al., 2021; Cisek & Kalaska, 2010). One useful approach to tackle this question is to study behavior in conflict tasks, where participants are presented not only with relevant but also with distracting and potentially conflicting information (e.g., Stroop, 1935; Eriksen & Eriksen, 1974; Simon & Rudell, 1967). Findings from conflict task studies have shown that distracting information affects not only decision-processes involved in selecting a response, but also motor processes involved in initiating and executing a response (e.g., Servant et al., 2016; Freud et al., 2015; Buetti & Kerzel, 2009). There is still uncertainty, however, about whether and how motor processes are involved when making actions under conflict. In the present study, we aim to provide some further insights into the role of motor processes in conflict processing by investigating how increased motor processing demands influence the effect of distracting information in the Simon task with mouse movements. As elaborated in more detail within our introduction, we reasoned that the Simon effect may be paradoxically reduced with larger motor demands, because target-related stronger activity during premotor and/or motor processing could counteract distractor-based activation.

In a standard visual Simon task, participants are required to make a left or right response to the identity of a lateralized target (e.g., a letter H or S) while ignoring its distracting spatial location (e.g., Hommel, 1994b; Lien & Proctor, 2000; Bausenhart et al., 2021; Hommel, 2011). Responses are typically faster and more accurate when target and response location are on the same (congruent trials) compared to opposite sides (incongruent trials). This so-called Simon effect has most often been observed when responses are simple key presses with the fingers of the left and right hand (e.g., Lien & Proctor, 2000; Hübner & Mishra, 2016; Mittelstädt et al., 2022). However, the effect can also be reliably measured when participants use other response effectors—vocal (e.g., Treccani et al., 2017; Wühr & Ansorge, 2007), eye (e.g., Leuthold & Schröter, 2006) and foot (e.g., Janczyk & Leuthold, 2017; Miller, 2016) responses—or perform more complex, continuous movements like reaching towards left versus right response boards (e.g., Salzer & Friedman, 2020; Finkbeiner & Heathcote, 2016), or moving a mouse cursor to response boxes presented on the left versus right side of the screen (e.g., Scherbaum et al., 2010; Grage et al., 2019; Wirth et al., 2020).

Many theoretical accounts of the Simon effect and other conflict effects assume that target-based information undergoes controlled processing within one route, whereas distractor-based information is processed presumably rather automatically by another parallel route (e.g., Eimer et al., 1995; Ridderinkhof et al., 1995; De Jong et al., 1994; Hübner et al., 2010; Ulrich et al., 2015; Wühr & Heuer, 2018; Kornblum et al., 1990). In essence, conflict effects emerge because distractor-based activation spills over to decision-making that is mainly driven by target-based activation and this activation superimposition improves (congruent trials) or impairs (incongruent trials) task performance. These accounts generally agree that activations are superimposed when selecting a response during decision-making. For example, a recently introduced model of conflict processing, the Diffusion Model for Conflict Tasks (DMC), assumes that the total response time (RT) in a trial is the result of a decision process in which activations are superimposed plus “the residual duration of all processes outside the decision process (e.g., stimulus encoding and response execution)” (p. 153 Ulrich et al. (2015))^1^.

In line with these accounts, many empirical findings suggest that Simon effects emerge at the stage in which the response is selected (e.g., Lu & Proctor, 1994; Masaki et al., 2000; Scerrati et al., 2017; Rubichi & Pellicano, 2004; Rubichi et al., 2000). For example, the Simon effect is reduced with higher cognitive load suggesting that distractor-based activation taps limited working memory capacity (e.g., Wühr & Biebl, 2011; Zhao et al., 2010). Furthermore, the Simon effect is modulated by mental task-sets—that is, the specific instruction required to translate a stimulus into a response (e.g., Metzker & Dreisbach, 2009; Theeuwes et al., 2014; Cohen et al., 2008; Hommel, 1993a).

Interestingly, however, there is also evidence that motor processes are involved in conflict processing (e.g., Lim & Cho, 2021; Buetti & Kerzel, 2009; Scorolli et al., 2015; Stürmer & Leuthold, 2003; Treccani et al., 2018; Freud et al., 2015; Miller & Roüast, 2016; Hietanen & Rämä, 1995; Hasbroucq et al., 2001). For example, EEG and EMG measures indicate that distracting information triggers motoric activation that can compete with motor activation provided by on-going decision processes (e.g., Servant et al., 2016; Stürmer et al., 2002). Note that these findings do not necessarily imply that distracting information only affects motor processes in parallel and independently from decision processes, because it is also possible that distractors produce motor activation after triggering cognitive-based response codes (cf. Valle-Inclán & Redondo, 1998; Hommel et al., 2004). Relatedly, it is similarly possible that independent Simon effects arise at both response selection and motor programming stages (e.g., Buetti & Kerzel, 2009, 2008). In any case, there are good reasons to assume that the competition between distractor-based and target-based activation might be localized during both premotor and motor processing, and that control processes also operate on motor processes.

The goal of the present study was to examine a novel approach to elaborate on how predictable motor processing demands modulate the superimposition of activation. While some studies suggest that anticipating motor demands can influence decision-making and/or motor processing (cf. Hagura et al., 2017; Marcos et al., 2015; Morel et al., 2017; Cos, 2017), it is unclear whether and how motor demands affect performance in the presence of distracting information. To tackle this issue, we used a visual Simon task with mouse movements and compared the Simon effect in blocks in which participants had to move a mouse cursor to either large versus small (Experiment 1) or near versus far (Experiment 2) response boxes presented on the screen. In general, we reasoned that increased action control in blocks associated with high (i.e., small or far responses boxes) compared to low (i.e., large or near responses) motor demands would result in amplified target processing since participants can anticipate that more demanding movements are required to reach the action goal. Thus, motor demands could bias processing at premotor and/or motor stages of processing. Assuming that the distractor and target processes are combined when a response is selected and/or a motor response is initiated, stronger target-based activation at the stage(s) where activation-superimposition occur(s) should lead to a reduced Simon effect under high compared to low motor demands. As will be considered in the General Discussion, there are also other possibilities regarding how increased motor demands may influence the Simon effect, but for now, we focus on this simplified biased competition account.

Critically, the temporal dynamics of conflict effects (including the Simon effect) make it difficult to infer the effects of experimental manipulations when looking only at mean RTs (e.g., Mittelstädt & Miller, 2020; Hommel, 1993b, 1995). For example, the visual Simon effect with horizontal key press responses is usually larger for faster than for slower responses as becomes evident from distributional analyses (e.g., Burle et al., 2013; De Jong et al., 1994; Luo & Proctor, 2020; Proctor et al., 2011; Wiegand & Wascher, 2005; Wascher et al., 2001). Specifically, delta plots display the size of conflict effects as a function of response speed by plotting the difference between congruent and incongruent mean response times (RTs) separately at RT percentile ranging from fastest to the slowest RTs (e.g., 10%, 20%, 30%). The slope of delta plots is usually interpreted as a marker of the time-course of distractor-based activation and, as illustrated in Fig. 1A, is primarily decreasing in the horizontal Simon task (e.g., Ridderinkhof, 2002; De Jong et al., 1994; Ellinghaus et al., 2017). Thus, manipulations which prolong processing duration can simply reduce the mean Simon effect because location-based activation has more time to fade out (cf., Hommel, 1994a, b; Mittelstädt et al., 2021).

To see whether the motor demand manipulations produce effects beyond those explainable purely in terms of time-varying distractor processing, we compared the delta plots in the low to the high motor demand condition (cf. Mittelstädt & Miller, 2020, 2018). Specifically, an overlapping delta plot pattern would indicate that the effects can be explained based purely on the unfolding of distractor-based activation (cf. solid and dashed delta plots in idealized prediction Fig. 1A). However, the Simon effect might be reduced beyond what can be explained by response speed which should shift the delta plot of the high demand condition downward relative to the delta plot of the low demand condition (cf. solid and dotted delta plots in Fig. 1A). It should be noted that decreasing Simon effects are primarily observed in the visual Simon task with key presses (e.g., Mittelstädt & Miller, 2020) and touch-based finger movements (e.g., Buetti & Kerzel, 2009), but analogous reasoning also applies when we would observe other time-varying characteristics of Simon effects with mouse movements (e.g., increasing delta plots, cf. Fig. 1B). Furthermore, motor demands may also affect the time-course of distractor-based processing and hence the slope of delta plots. Thus, the general point is that interpretations based solely on mean RT may not be sufficient to rule out accounts in which the motor demand manipulation influences the Simon effect exclusively because of time-varying activations.

The present manipulation of motor demands is motivated by Fitt’s Law (Fitts, 1954), according to which the difficulty of the motor task increases with the distance to the target and with decreasing target size. Thus, the manipulation of target size (Experiment 1) and of target distance (Experiment 2) should both affect movement time (MT)^2^.

**Fig. 1:**
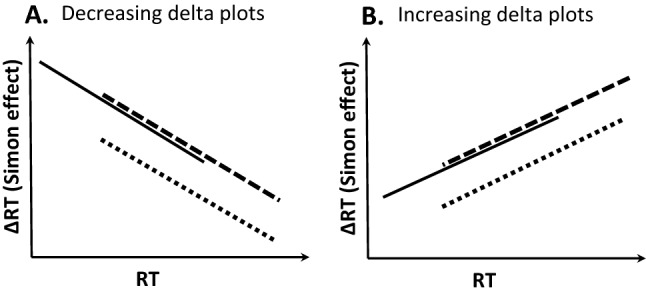
Schematic depiction of two qualitatively different delta plots shifts of a high (slow) demand motor processing condition (i.e., dashed and dotted delta plots) compared to a low (fast) demand condition (i.e., solid delta plots) separately for generally decreasing (**A**) or increasing delta plots (**B**)

## Experiment 1

In the first experiment, we manipulated motor demands by reducing the size of the response boxes. Thus, in different block of trials, the response boxes were either large (low motor demand) or small (high motor demand).

### Methods

#### Participants

30 people were tested online. Data of three participants were excluded due to moving the mouse out of the starting box region before stimulus onset in over 25% of trials (for more details, see data preparation section). The final sample consisted of 27 participants (21 female, all right-handed), ranging in age from 19 to 28 years (*M* = 21.93)^3^. All participants gave informed consent, were tested in a single session lasting approximately 35 min, and received course credits for participation.

#### Apparatus and stimuli

**Fig. 2:**
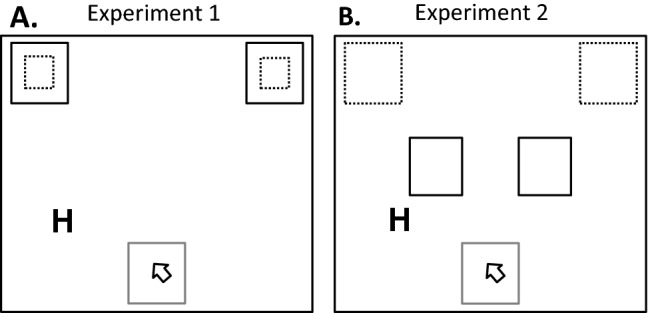
Schematic illustration of the stimulus display in Experiment 1 (**A**) and Experiment 2 (**B**). Participants had to initiate each trial by clicking into the starting box (depicted as grey squares) and after 500 ms a target letter was presented to the left or right of the screen. Participants responded by clicking into one of the two response boxes (depicted as black squares). Response boxes with solid lines were used in low motor demand blocks. Response boxes with dotted lines were used in high motor demand blocks. In Experiment 1, the size of response boxes differed by factor 3 and in Experiment 2, the distance of response boxes differed by factor 2

The experiment was conducted online using the JavaScript library jsPsych (e.g., De Leeuw, 2015), by extending the mouse-plugin reported in Schütt et al. (2022). All visual stimuli were presented in black on a grey background. Figure 2A illustrates the stimulus display. The two stimulus letters (i.e., H and S) were randomly assigned to left- and right target responses. A starting box was presented in the center at the bottom of the screen. Two response boxes were presented to left and right upper screen positions. In high motor demand blocks, the size of response boxes was reduced by factor 2. Initiation times were calculated from the time of stimulus onset until participants left the starting box. The remaining time (i.e., until participants made a click with the mouse in a response box region) was considered movement times. Thus, overall response times reflect the sum of initiation and movement times. Note that in both experiments we also reanalyzed movement times while excluding trials in which participants paused their movements (with pauses defined as no movement for more than 50 ms during the interval between movement onset and clicking in or reaching the target region). The movement time results on both mean and distributional RT level were similar to the ones reported in the present result sections when excluding these trials, indicating that pause-and-restart movements are very unlikely to have contaminated the reported findings.

#### Procedure

Motor demands (low vs. high) were held constant within a block and alternated across sequential blocks. Half of the participants were tested with a block with low motor demand for the first block. The experiment consisted of ten blocks of trials, of which the first two were considered practice blocks and were removed from subsequent analyses. The practice blocks consisted of 20 trials each, whilst the remaining blocks consisted of 64 trials each. Participants were instructed to initiate each trial by clicking the left mouse button within the starting box region, after which a fixation cross appeared on the screen for 500 ms. Following the offset of the fixation cross, a single letter was presented to the left or right side of the screen (i.e., Simon task). We opted to display targets below response areas—and not within response areas—to be consistent with other (Simon) mouse-tracking studies (e.g., Scherbaum et al., 2010; Grage et al., 2019; Scherbaum & Kieslich, 2018; Scherbaum & Dshemuchadse, 2019) and to minimize effects not related to motor demands (e.g., on perceptual components) as much as possible. The letter remained on the screen until participants responded (i.e., no response deadline) by clicking into the left or right response box. Feedback was displayed for either 1 s or (correct) or for 2.5 s (error) before the next trial started.

#### Data preparation

For both percentage error (PE) and time analyses (i.e., initiation times and movement times) in both experiments, we made sure that mouse movements were continuously recorded and we excluded trials with corrupt trajectories. This lead to the exclusion of 5 (< 0.01%) and 20 (< 0.01%) trials in Experiment 1 and 2, respectively. Then, data of participants who failed to follow task instructions by moving the cursor out of the starting box region before the stimulus letter was presented (*n* = 3, with 98%, 94% and 33% of trials, respectively). For the remaining 27 participants, less than 2.5% of trials were removed due to this reason. Based on visual inspection of the overall response time distribution, we then additionally excluded “too-fast” (< 50 ms, < 0.5%) and “too-slow” (> 4 s, < 0.1%) trials. For time analyses, we additionally excluded choice error trials (< 1%). In both experiments, similar results were also obtained when including time outliers. Moreover, we also analyzed the data in the two experiments using (a) a stricter “too-fast” criterion (i.e., up to 200 ms) which is commonly used in key-based reaction time experiments to exclude anticipatory trials and (b) stricter “too-slow” criteria (i.e., 2 s and 3 s). The result pattern and test statistics were quite similar, suggesting that the motor manipulation does not solely affect processes taking place immediately after stimulus onset.

#### Design

For the analyses on mean initiation times, mean movement times and mean PE as dependent variables, we performed repeated-measures ANOVAs with the within-subject factors of motor demands (low, high) and congruency (congruent, incongruent). For the analyses on distributional times, we constructed delta plots separately for low and high motor processing blocks by creating 9 time percentiles (i.e., 10%, 20%,...) separately for each participant within each of four conditions (i.e., low/high $\times$ congruent/incongruent). Very similar results were also obtained in analyses using four percentiles. In order to further compare the shapes and offset of the two delta plots, we summarized the delta plot for each participant and condition with a linear regression model predicting the delta in each bin from the mean time in that bin (e.g., Pratte et al., 2010; Mittelstädt & Miller, 2020, 2018). To check for an offset between the two conditions, we used the regression model for each condition to compute the predicted Simon effect at each participant’s individual mean initiation and/or movement time. Thus, this analysis allowed us to compare the Simon effects at a common time value thereby controlling for potential time-based fluctuations of the size of the Simon effect. We then performed paired *t*-tests on slopes and predicted Simon effects in order to test for differences in the time-course and offset of delta plots between the two conditions (e.g., Mittelstädt & Miller, 2020; Ellinghaus & Miller, 2018; Hübner & Töbel, 2019; Mackenzie et al., 2022).

### Results and discussion

#### Initiation times (ITs)

**Fig. 3:**
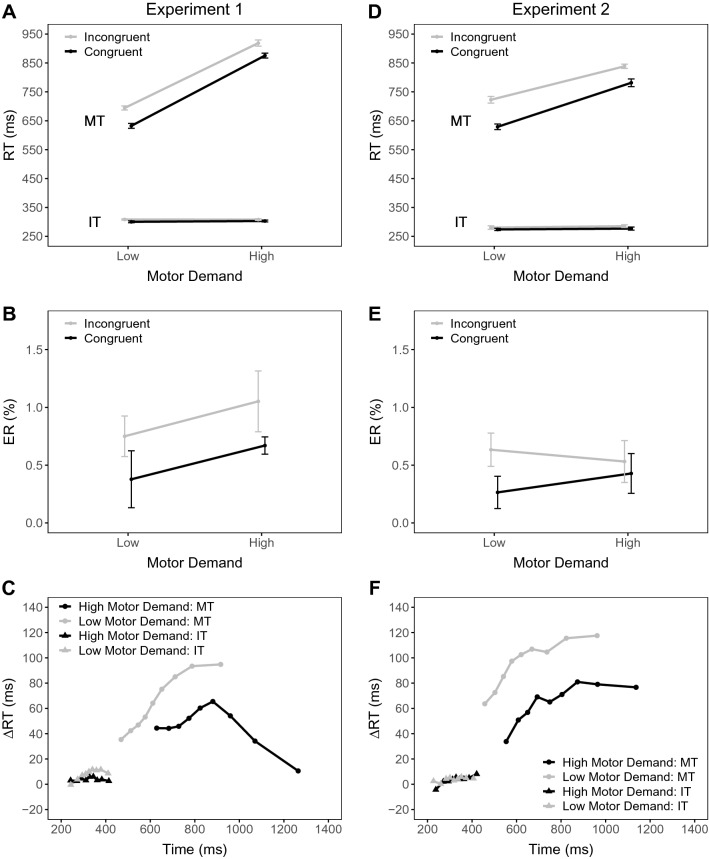
Panels **A**, **B**, **D** and **E** show mean initiation time (IT), mean movement time (MT) and mean percentage errors (PE) as a function of motor demands (low, high) and congruency (congruent, incongruent) separately for Experiments 1 and 2. Panels **C** and **F** show delta plots showing incongruent minus congruent differences in mean times (IT and MT) within each of 9 time deciles, plotted against the decile average times, as a function of motor demand (low, high) separately for Experiments 1 and 2. The error bars represent 95% within-participant standard errors calculated according to Morey (2008)

Figure 3A shows the mean ITs as a function of motor demands (low, high) and congruency (congruent, incongruent). As can be seen from this figure, the ITs were quite similar across conditions and the ANOVA only revealed a significant main effect of congruency, *F*(1, 26) = 7.25, *p* = 0.012, $\eta ^2_p$ = 0.22 (with all other $p \mathrm{s} > 0.564$, all $\eta ^2_p$s < 0.02). The mean ITs were smaller in congruent than incongruent trials (302 ms versus 309 ms). The IT delta plots for the two motor demand conditions shown in Fig. 3C not only had similar shapes but also overlapped across the whole IT distribution. The mean slopes were positive for both low (0.07) and high (0.02) motor demands and a paired *t*-test indicated no significant difference, *t*(26) = 1.28, *p* = 0.212, *d* = 0.25. Furthermore, there was evidence for an offset between the two delta plots as indicated by a significant difference between the predicted Simon effects for the low (7 ms) and high (2 ms) motor demand conditions, *t*(26) = 1.71, *p* = 0.010, *d* = 0.33.

#### Movement times (MTs)

Figure 3A also shows the corresponding mean MTs. The ANOVA revealed significant main effects of motor demands, *F*(1, 26) = 430.24, $p<$ 0.001, $\eta ^2_p$ = 0.94, and congruency, *F*(1, 26) = 32.32, $p<$ 0.001, $\eta ^2_p$ = 0.55. The mean MT was smaller in blocks with low than high motor demands (659 ms versus 893 ms), and the mean MT was also smaller in congruent than in incongruent trials (749 ms versus 802 ms). There was also a significant interaction reflecting a larger Simon effect with low (62 ms) than high (43 ms) demands, *F*(1, 26) = 5.11, *p* = 0.032, $\eta ^2_p$ = 0.16.

As can be seen in Fig. 3C, the delta plots in the low and high demand conditions seem to follow qualitatively distinct time-courses—that is, only the delta plot in the high demand condition showed a decreasing time-course for larger MTs. Critically, across the entire range of MTs, the Simon effect in high demand blocks was consistently less than the one observed in low demand blocks. The mean slope was positive for the low demand condition and negative for the high demand condition (i.e., 0.15 and − 0.04, respectively), and this difference was significant, *t*(26) = 4.54, $p<$ 0.001, *d* = 0.87. Most importantly, the predicted Simon effect was larger for the low (85 ms) than high demand condition (49 ms), and a paired *t*-test indicated a significant difference between these values, *t*(26) = 5.74, $p<$ 0.001, *d* = 1.11. Thus, increased motor demands reduced the Simon effect in MTs by more than can be explained by the time-course of location-based activation. For completeness, we also reanalyzed the data while considering overall reaction times (i.e., initiation times + movement times). As can be seen in Appendix A, the results of this analysis also revealed smaller Simon effects under high compared to low motor demands on both mean and distributional RT level.

#### Percentage errors (PEs)

Overall, mean PEs were quite low (< 1%) and the descriptive pattern was generally consistent with the one found for mean RTs (see Fig. 3B). The ANOVA revealed no significant effects (all $p \mathrm{s}>$ 0.100, all $\eta ^2_p \mathrm{s}<$ 0.11).

## Experiment 2

In the second experiment, we manipulated motor demands by varying the distance between the start region and the response box regions. Thus, in different block of trials, the response boxes were in either a near (low motor demands) or far (high motor demands) distance from the starting box.

### Methods

#### Participants

Another sample of 30 participants from the same participant pool were tested online. Using the same trial exclusion criterion described for Experiment 1, the data of six participants were excluded. The final sample consisted of 24 participants (18 female, 23 right-handed), ranging in age from 19 to 23 years (*M* = 20.62). All participants gave informed consent, were tested in a single session lasting approximately 35 min, and received course credits for participation.

#### Apparatus, stimuli and procedure

The apparatus, stimuli and procedure were the same as in Experiment 1 except for the following changes. The response box always had the same size and instead motor demands were manipulated by varying the distance from the starting box (cf. Fig. 2B).

#### Data preparation and design

We first excluded the data of one participant due to a technical error and we then followed the same data preparation procedure and design as in Experiment 1. Specifically, we then excluded data of participants who moved the cursor out of the starting box region before the stimulus appeared in a large proportion of trials (*n* = 5, with 93%, 81%, 73%, 68% and 27% of trials, respectively). For the remaining 24 participants less than 3% of trials were excluded due to this reason. The first two blocks were considered practice and excluded from any analyses. For both PE and time analyses, we excluded “too-fast” (< 50 ms, < 0.5%) and “too-slow” (> 4s, < 0.2%) trials. For time analyses, we additionally excluded choice error trials (< 1%).

### Results and discussion

#### Initiation times (ITs)

Figure 3D shows the mean ITs as a function of the experimental factors. The ANOVA with the within-subject factors of motor demands and congruency revealed again only a significant main effect of congruency, *F*(1, 23) = 6.21, *p* = 0.026, $\eta ^2_p$ = 0.20. (all other $p \mathrm{s}>$ 0.569, all $\eta ^2_p s<$ 0.02). The mean IT was smaller in congruent than in incongruent trials (275 ms versus 283 ms). The delta plots were overlapping with a similar shape (Fig. 3F). Indeed, there was no significant difference between the slopes in the low (0.03) and high (0.05) motor demand condition, *t*(23) = 0.61, *p* = 0.548, *d* = 0.12. Furthermore, there was no significant difference between the predicted Simon effects at the same absolute mean ITs in the low (3 ms) and high (1 ms) motor demand condition, *t*(23) = 0.52, *p* = 0.607, *d* = 0.11.

#### Movement times (MTs)

Figure 3D shows the mean MTs separately for each condition. The ANOVA with the within-subject factors of motor processing demands and congruency revealed again significant main effects of motor demands, *F*(1, 23) = 84.79, $p<$ 0.001, $\eta ^2_p$ = 0.79, and congruency, *F*(1, 23) = 65.97, $p<$ 0.001, $\eta ^2_p$ = 0.74. The mean MT was smaller in blocks with low than high motor demands (675 ms versus 810 ms), and the mean MT was also smaller in congruent than in incongruent trials (705 ms versus 780 ms). There was also a significant interaction reflecting a larger Simon effect with low (92 ms) than high (58 ms) demands, *F*(1, 23) = 7.24, *p* = 0.013, $\eta ^2_p$ = 0.24.

As can be seen in Fig. 3F, the delta plots in the two conditions followed similar, slightly increasing, time-courses. Most importantly, as in Experiment 1, the Simon effect in high demand blocks was consistently less than the one observed in low demand blocks across the whole MT distribution. Indeed, the mean slopes were positive for both the low and high demand conditions (i.e., 0.11 and 0.08, respectively), and a paired *t*-test indicated no significant difference between these values, *t*(23) = 0.66, *p* = 0.514, *d* = 0.14. Furthermore, the predicted Simon effect was significantly larger for the low (103 ms) than high demand condition (64 ms) at the same absolute MTs, *t*(23) = 3.58, *p* = 0.002, *d* = 0.73. Thus, these linear-fit-based comparisons confirm the visual inspection regarding the conclusion that the Simon effect in MTs is larger for low than high motor demand when controlling for the increasing time-course of this effect. The results of the overall RT analysis in Appendix A lead to the same conclusion.

#### Percentage errors (PEs)

Again, the mean PEs were quite low (0.46%). The pattern was generally quite similar to the one found on mean MTs (see Fig. 3E). There was a significant main effect of congruency, reflecting smaller mean PE in congruent than in incongruent trials (0.35% versus 0.58%), *F*(1, 23) = 5.23, *p* = 0.031, $\eta ^2_p$ = 0.19. The interaction was also significant, *F*(1, 23) = 6.44, *p* = 0.018, $\eta ^2_p$ = 0.22. The Simon effect in PE was larger with low (0.37%) than high processing demands (0.10%) (with *p* = 0.661, $\eta ^2_p$ = 0.01 for the main effect of motor demands).

## General discussion

In the present study, we examined the effect of increasing the motor processing demands on conflict processing in the Simon task. Specifically, we compared Simon effects in blocks that required more versus less precise mouse movements (i.e., small vs. large response boxes in Exp. 1) and in blocks that required long versus short mouse movements (i.e., far versus near responses boxes in Exp. 2). We reasoned that participants would increase action control by strengthening target-related activation in blocks with high versus low motor demands and that this would reduce the distracting effect of location-based activation. In line with this hypothesis, the Simon effects on mean movement times were reduced under high motor demands and additional delta plot analyses revealed that this pattern holds true even when controlling for time-varying distractor-based activation.

In general, the present results fit well to studies emphasizing the need to consider motor processes when studying perceptual decision-making (e.g., Pierrieau et al., 2021; Cisek & Kalaska, 2005; Ulrich et al., 2007; Servant et al., 2021; Donner et al., 2009; Selen et al., 2012). Thus, while formal decision-making models often (at least implicitly) assume that control processes operate independently from motor processes, the present results favor accounts that emphasize the interaction of cognitive control and motor planning (e.g., Wolpert & Landy, 2012; Wispinski et al., 2018). For example, researchers have shown that when making decisions under conflicting sources of information, the distracting activation at least partially also impacts on motor processes involved in initiating and executing the selected responses (e.g., Weissman, 2019; Buetti & Kerzel, 2009; Servant et al., 2016; Freud et al., 2015). Critically, we extend these previous findings by showing that directly *manipulating* motor processes can recursively bias conflict processing.

This bias could be explained by a purely motor-based account: Assuming that participants more strongly activate the target-based motor responses when a high level of motor demands is required, this would reduce the contribution of distractor-based motor activation when the two activations superimpose. The finding that the effects of the motor manipulation were primarily reflected in movement times rather than in initiation times reinforces the idea of differential activations under high versus low motor demands within motor-related stages. Although speculative, analogous reasoning may also explain why the Simon effect was larger with feet than hands responses in an earlier study (Mittelstädt & Miller, 2020). Since we are often required to perform more precise movements with our hands than feet, hand-related motor activation is probably better shielded from the influence of distractor-based activation.

However, it is also possible that a high level of motor demands may tap on more of the limited central resources involved in selecting a response at a premotor level (for similar suggestions, see e.g., (Ulrich et al., 2007; Park et al., 2021; Welch, 1898). If so, one would intuitively expect that the Simon effect would tend to be larger instead of smaller for high than for low motor demands—also considering that many conflict effects (e.g., Stroop, Eriksen flanker) usually increase in size under cognitive load (e.g., Lavie et al., 2004). Interestingly, however, the Simon effect actually decreases under cognitively more demanding conditions (e.g., Wühr & Biebl, 2011; Zhao et al., 2010), with the delta plot pattern resembling the one found in the present study (e.g., Mittelstädt & Miller, 2020). One may speculate that less of the central resources (e.g., working memory) are devoted to distractor-based processing not only when cognitive load (e.g., Wühr & Biebl, 2011) BUT also when motor load increases. Relatedly, more efficient central (premotor) target processing with high motor demands might also entirely explain—or at least partially contribute to—modulations of the Simon effect^4^. To separate influences on premotor versus motor processing, it might be possible to localize the effects of the present manipulation with psychophysiological measures (e.g., lateralized readiness potential, see e.g., (Leuthold, 2011; Mittelstädt et al., 2022).

It might also be useful to extend the current approach of investigating the effects of motor demands on conflict processing to other versions of conflict tasks and motor demand manipulations (e.g., manipulating the force required to press a key when using response force-sensitive keys; cf. (Mattes et al., 2002; Miller & Alderton, 2006). While the central results regarding Simon effects in movement times were generally consistent across the present experiments, there are also some hints that the effects of the experiment-specific motor demand manipulations (i.e, response box sizes versus distance) on processes modulating the Simon effect might at least partially differ. For example, the delta plots showed a decreasing time-course for larger movements times (i.e., > 900 ms) in Experiment 1 but not Experiment 2. Assuming that the slope of delta plots capture inhibitory processes (e.g., Ridderinkhof, 2002), this may indicate the presence of some extra suppression-related control processes operating on distractor-based activation when the size of response boxes become smaller.^5^ Furthermore, as can be seen in the Appendix B, exploratory analyses of the mouse *trajectory* data also point to both shared and distinct influences of the specific motor manipulations. Specifically, in both experiments mean deviations in mouse trajectories were smaller when motor demands increased which seem to reinforce the idea of better motor control within high compared to low motor demand blocks. Moreover, in both experiments, the mouse trajectories were also susceptible to the distracting influences of stimulus location. Interestingly, however, this trajectory-based mean Simon effect was smaller when motor demands increased in Experiment 1, whereas this effect actually increased with motor demands in Experiment 2.

In any case, even though the results do not allow decisive evidence regarding whether the motor manipulation interacts with premotor response selection and/or motor response activation in the specific experiments, the manipulation clearly influenced conflict processing throughout the entire movement time distribution in both experiments. Thus, the results are generally consistent with the idea that motor demands can bias the activation-competition process which is implemented in conflict-task models like DMC (e.g., Ulrich et al., 2015). In order to more directly examine this possibility, we examined whether and how DMC captured the empirical result pattern found in the two experiments. As can be seen in Appendix C, the model was generally able to capture the observed data with changes in estimated parameter values that were quite consistent across experiments. Most important, distractor-based activation was reduced in the high motor demand condition (i.e., the strength of the amplitude parameter of the distractor process was smaller with high than low motor demands). Although it also seems plausible that non-decision time increased under high motor demands, it should be emphasized that evidence accumulation models like DMC do not specify whether and how control processes are involved in non-decision (e.g., motor) processing. Therefore, some caution needs to be applied when interpreting these exploratory fitting results (e.g., Roberts and Pashler, 2000) and the comparison with (and development of) computational conflict-task models that bridge both cognitive and motor control systems is clearly warranted. We hope that the central empirical finding of reduced conflict effects with higher motor demands will help tackle this issue.

## Figures and Tables

**Fig. 4:**
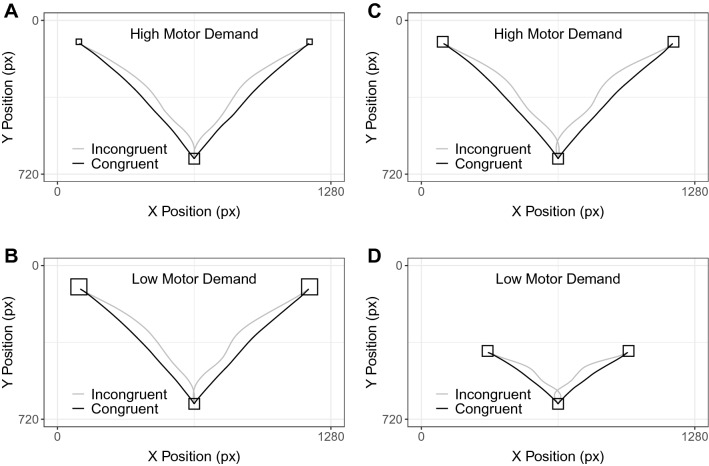
The lines show the average mouse trajectories for trials requiring left vs. right responses as a function of motor processing demands (low, high) and congruency (congruent, incongruent) for Experiments 1 and 2

**Fig. 5:**
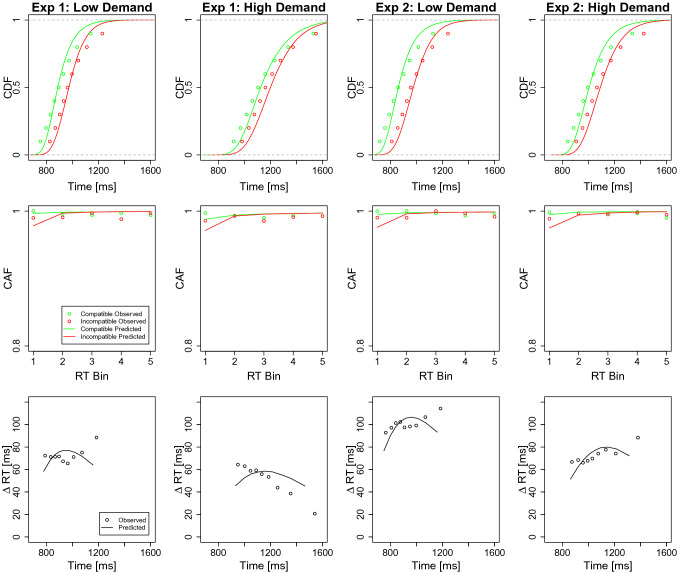
Experimental results and predictions of the Diffusion Model for Conflict (DMC) Tasks. The panels within each column depict the fitting results of one of the two conditions (i.e., high vs. low motor demands) separately for Experiment 1 and Experiment 2. The panels within each row depicts the cumulative distribution function (CDF) of correct RTs separately for congruent and incongruent trials, conditional accuracy functions (CAF) separately for congruent and incongruent trials, RT delta plots showing incongruent minus congruent differences in mean RTs within each of 9 deciles plotted against the decile averages, respectively

## Additional analyses regarding overall reaction times

In this appendix, we present the analyses on overall reaction times (i.e., initiation times plus movement times).

### Experiment 1

The ANOVA revealed significant main effects of motor demands, *F*(1, 26) = 467.18, $p<$ 0.001, $\eta ^2_p$ = 0.95, and congruency, *F*(1, 26) = 42.33, $p<$ 0.001, $\eta ^2_p$ = 0.62. The mean RT was smaller in blocks with low than high motor demands (968 ms versus 1203 ms), and the mean RT was also smaller in congruent than in incongruent trials (1056 ms versus 1114 ms). There was also a significant interaction reflecting a larger Simon effect with low (70 ms) than high (48 ms) demands, *F*(1, 26) = 6.08, *p* = 0.021, $\eta ^2_p$ = 0.19.

The mean slope was slightly positive for the low demand conditions and negative for the high demand condition (i.e., 0.02 and − 0.06, respectively), but this difference was not significant, *t*(26) = 1.56, *p* = 0.131, *d* = 0.30. The predicted Simon effect was larger for the low (77 ms) than high demand condition (55 ms), and a paired *t*-test indicated a significant difference between these values, *t*(26) = 3.01, $p<$ 0.001, *d* = 0.58.

### Experiment 2

The ANOVA with the within-subject factors of motor demands and congruency revealed again significant main effects of motor demands, *F*(1, 23) = 105.53, $p<$ 0.001, $\eta ^2_p$ = 0.82, and congruency, *F*(1, 23) = 100.48, $p<$ 0.001, $\eta ^2_p$ = 0.81. The mean RT was smaller in blocks with low than high motor demands (953 ms versus 1091 ms), and the mean RT was also smaller in congruent than in incongruent trials (980 ms versus 1063 ms). There was also a significant interaction reflecting a larger Simon effect with low (103 ms) than high (66 ms) demands, *F*(1, 23) = 6.29, *p* = 0.020, $\eta ^2_p$ = 0.21.

The mean slopes were positive for both the low and high demand (i.e., 0.04 and 0.05, respectively), and a paired *t*-test indicated no significant difference between these values, *t*(23) = 0.62, *p* = 0.537, *d* = 0.13. Furthermore, the predicted Simon effect was significantly larger for the low (143 ms) than high demand condition (87 ms) at the same absolute RTs, *t*(23) = 2.16, *p* = 0.041, *d* = 0.44.

## Additional analyses regarding mouse trajectories

In this appendix, we present the analyses on mouse trajectories. Specifically, we explored how strongly participants’ mouse trajectories deviated from an optimal path as a function of the experimental conditions by using the R package mousetrap (Kieslich & Henninger, 2017). For this purpose, we calculated per participant, the difference between optimal and observed trajectories across each trial (measured at 101 points) for the maximum the corresponding average deviation within each of four conditions (i.e., low/high $\times$ congruent/incongruent). We then performed a repeated-measures ANOVA with the within-subject factors of motor processing demands (low, high) and congruency (congruent, incongruent) on the mean deviations.

### Experiment 1

Figure 4A &B show the mouse trajectories as function of motor processing demands (low, high). The ANOVA revealed significant main effects of motor processing demands, *F*(1, 26) = 7.91, *p* = 0.009, $\eta ^2_p$ = 0.23, and congruency, *F*(1, 26) = 71.81, $p<$ 0.001, $\eta ^2_p$ = 0.73. The mean deviation was smaller in blocks with high than low motor processing demands (25 vs. 28 px), suggesting that participants movements became more optimal when motor difficulty increased. The mean deviation was also smaller in congruent than in incongruent trials (13 vs. 40 px), indicating that Simon effects were also present in mouse trajectories. There was also an interaction between motor processing demands and congruency, *F*(1, 26) = 15.35, *p* = 0.001, $\eta ^2_p$ = 0.37. The distracting influences of stimulus location on mouse trajectories were smaller in high (25 px) than low demand blocks (32 px).

### Experiment 2

Figure 4C,D shows the mouse trajectories as function of motor processing demands (low, high). The results of the ANOVA on mean deviation trajectories revealed again that all effects were significant. The main effect of motor processing demands indicated smaller deviation in blocks with high than low demands (21 versus 29 px), *F*(1, 23) = 25.63, $p<$ 0.001, $\eta ^2_p$ = 0.53. The main effect of congruency indicated smaller deviations in congruent and in incongruent trials (11 versus 39 px), *F*(1, 23) = 115.66, $p<$ 0.001, $\eta ^2_p$ = 0.83. In contrast to Experiment 1, the significant interaction reflected a reduced trajectory-based Simon effect with low (24 px) than high (32 px) demands, *F*(1, 23) = 15.09, *p* = 0.001, $\eta ^2_p$ = 0.40.

## Additional information regarding DMC model fitting

The DMC model assumes that the outputs of controlled (target-based activation) and automatic (distractor-based activation) processes are superimposed into a single Wiener diffusion process (with the diffusion constant $\sigma$) toward the correct decision boundary *b*. The drift rate of this superimposed diffusion process is calculated based on the sum from the temporally constant input of a target-based process with drift rate $\mu _{c}$ and the time-varying input of a distractor-based process with drift rate $\mu _{i}(t)$. Specifically, the input from the distractor-based process is modeled as a pulse-like gamma density function with shape parameter *a* which reaches its peak amplitude *A* at time $\textit{t}_{peak}= (a-1)\cdot \tau$ after which it decreases back to zero. RT in a given trial is the sum of the decision time needed to reach the response boundary *b* plus a normally distributed non-decision (residual) time (i.e., with $\mu _{R}$ and $\sigma _{R}$). Starting point variability is implemented by sampling from a beta-shaped distribution *B* which varies symmetrically around zero from $\textit{b}_{1}$ to $\textit{b}_{2}$.

The DMC model was fitted to the observed individual data (i.e., overall reaction times) of the two experimental conditions (i.e., high vs. low motor processing demands) from each experiment by using the R-package DMCfun (Mackenzie & Dudschig, 2021). Following (Ulrich et al., 2015), the model was fitted simultaneously to condition-specific errors and RT distributions by minimizing the root-mean-squared error (RMSE) between observed and predicted values (see also (Mittelstädt et al., 2021)). Specifically, the DMCfun package calculates a cost value for both the percentile RT data (RMSE_RT_) and error data (RMSE_CAF_) with the total cost being a weighted sum of the two (for more details, see (Mackenzie & Dudschig, 2021; Ulrich et al., 2015)) As fitting algorithms, DMCfun makes use of the R-package DEoptim (Mullen et al., 2011) which uses the differential evolution algorithm.

The mean best-fitting parameters and mean RMSEs as a function of motor demands for each experiment are shown in Table 1, and the corresponding model fits to capture the distributional RT and error data are visualized in Fig. 5. In the following, we report the results of paired-tests with the factor motor demands (low, high) on the estimated values.

### Experiment 1

The strength of distractor-based processing (i.e., amplitude $\textit{A}$) was reduced under high compared to low demands, *t*(26) = 5.25, $p<$ 0.001. Furthermore, the drift rate of target-based processing $\mu _{c}$ was smaller under high compared to low demands, *t*(26) = 7.99, $p<$ 0.001. Both mean and variability of residual times were larger under high compared to low demands with *t*(26) = 11.46, $p<$ 0.001, and, *t*(26) = 3.21, *p* = 0.003, respectively. There were no significant differences concerning the other parameters (all *p*s > 0.356).

### Experiment 2

The result pattern was very similar to Experiment 1. Specifically, the amplitude $\textit{A}$ of the distractor-process was again reduced under high compared to low demands, *t*(23) = 3.26, *p* = 0.003. The drift rate of target-based processing $\mu _{c}$ was again smaller under high compared to low demands, *t*(23) = 2.83, *p* = 0.010. Finally, mean residual times were again larger under high compared to low demands, *t*(23) = 10.18, $p<$ 0.001. There were no significant differences concerning the other parameters (all *p*s > 0.195).

**Table 1:** Best-fitting parameters of the Diffusion Model for Conflict tasks (DMC) (Ulrich et al., 2015) to the experimental data of the two motor processing demand conditions within each experiment as well as weighted root-mean-square-errors (RMSE) averaged across participants

	Experiment 1		Experiment 2	
	Low	High	Low	High
DMC best-fitting parameters				
Amplitude $\textit{A}$ of distractor process	29.8 (2.6)	19.9 (19.7)	37 (4.0)	25 (2.5)
Peak (ms) $\tau$ of distractor process	213 (20)	230 (28)	261 (20)	278 (23)
Decision boundary $\textit{b}$	189 (6)	188 (7)	198 (8)	188 (7)
Drift rate $\mu _{c}$ of target process	0.66 (0.04)	0.46 (0.02)	0.61 (0.03)	0.56 (0.03)
Mean residual time (ms) $\mu _{R}$	659 (19)	786 (24)	613 (18)	729 (22)
Variability residual time (ms) $\sigma _{R}$	30 (4)	53 (6)	25 (4)	32 (7)
Shape $\alpha _{s}$ of starting point distribution	3.87 (0.02)	3.82 (0.04)	3.77 (0.05)	3.75 (0.06)
Goodness-of-fit (*RMSE*)	16.60 (1.95)	33.72 (4.54)	16.73 (2.14)	27.64 (4.68)

Standard Error (SE) of means in parentheses. Following (Ulrich et al., 2015), the step size was *t* = 1 ms, the diffusion constant was fixed at $\sigma$ =4, and the shape parameter of the distractor process was fixed at $\textit{a}$ = 2

## Footnotes

1 Although the model is phrased in terms of decision stage superimposition as is tradition with these kind of models, mathematically the superimposition could just as easily happen at some other stage, thus this assumption is not required. For example, Ulrich et al. (2015) have also mentioned that DMC is conceptually consistent with architectures where the two routes ”converge at the level of response activation, where overt responses are initiated and executed.“ (p. 152 Ulrich et al., (2015).

2 After completing the present study, we became aware of a study by Wirth et al. (2020) which explored the influence of several design parameters on measures of finger-tracking performance (e.g., initiation and movement times) in a Simon task-set up. Although this study was not designed to investigate how motor demands influence conflict processing, it is interesting to note that they also manipulated the size of the response areas in one experiment but this manipulation did not modulate the Simon effect in mean initiation nor mean movement times. Unfortunately, it is not clear whether this pattern holds true when considering the whole IT and MT distributions, because they did not report any delta plot analyses. Moreover, one aspect of their experimental procedure makes it generally difficult to derive any post-hoc conclusion regarding whether and how motor demands influences conflict processing: The color filling of one of the left versus right response areas served as the target-based information and hence, varying the size of response areas also varied the visibility of targets. Thus, there might also be effects on target and/or distractor processing due to the rather perceptual component of their manipulation. Similarly, it is difficult to interpret their post-hoc between-experiment comparison related to conditions with different distances of response areas in *finger-tracking*. Here, the authors report significantly smaller mean congruency effects in movement times between the near condition of their Exp. 1 and their far condition of Exp. 2 (no delta plots are reported). As the authors themselves concede (Wirth et al., 2020), however, the interpretation is hindered, since the impact of the other experiment-specific conditions on this comparison is unclear. In addition, there was surprisingly no evidence that the overall time to complete a trial differed between the respective conditions.

3 The sample size in the two experiments was somewhat arbitrarily set, but both practical constraints (e.g., participant availability) and empirical constraints (e.g., effect size in previous studies, larger variability in an online-setting) were taken into account. In a previous study (Experiment 4 in Mittelstädt and Miller 2020), we observed a rather large effect size (*d* = 1.12) for a shift between delta plots (as measured via paired t-test on predicted Simon effects as in the present study). With the actual sample sizes of 27 (Exp. 1) and 24 participants (Exp. 2) we would have over 80% power to detect a significant effect regarding the delta plot comparison of at least *d* = 0.50 (Exp. 1) and *d* = 0.53 (Exp. 2) at a significance level of $\alpha$ = 0.05 (one-sided paired *t*-test). The cut-off to exclude participants was somewhat arbitrarily set after inspecting the proportion of “too-early” trials for each participant in this and the second experiment. Note, however, that a qualitatively very similar result pattern and inferential statistics were also obtained when using more or less strict cut-offs (e.g., excluding participants with over 10 or 70% too-early trials).

4 Note that it may be possible that decision boundaries are higher in more demanding conditions. While this may contribute to the observed motor costs in movement times, it is not really clear how this would result in the modulation of the Simon effects. Furthermore, it should be also emphasized that there was no sign of a speed-accuracy trade-off across conditions, and no evidence of changes in decision boundaries in the exploratory fitting results. Thus, we consider it unlikely that strategic changes in boundaries can account for the present pattern.

5 We also reanalyzed all results by using an alternative measure of the time needed in each trial based on the trajectory velocity. Specifically, we calculated timepoints where movement velocity was first greater than a criterion (velocity onset) and second, when movement velocity was below this criterion and was near the response box zone (velocity offset). Velocity onsets (offsets) were determined by calculating a combined velocity profile from the *x*- and *y*- coordinates, with onsets (offsets) defined as the timepoint when velocity exceeded (fell below) 2 px/ms. We reasoned that with this analyses the time only captures movement times which reflect “ballistic” type movements towards the general response zone. This removes the remaining time portion of the movement involving small corrective type movements within the vicinity of the response zone, which was particularly evident when responding to the small response zones used in Experiment 1. Interestingly, the difference between Simon effects on delta plots when using this ballistic movement measure was only found in Experiment 2 but not Experiment 1 providing further support for especially late effects of the motor demand manipulation in Experiment 1.

## Declarations

### Open practice statements

Raw data are available via the Open Science Framework at https://osf.io/ce9hm/. Analyses scripts are available upon reasonable request.
